# Failure in Cognitive Suppression of Negative Affect in Adolescents with Generalized Anxiety Disorder

**DOI:** 10.1038/s41598-017-07063-5

**Published:** 2017-07-26

**Authors:** Dazhi Yin, Wenjing Liu, Kristina Zeljic, Qian Lv, Zhiwei Wang, Meina You, Weiwei Men, Mingxia Fan, Wenhong Cheng, Zheng Wang

**Affiliations:** 1 Institute of Neuroscience, State Key Laboratory of Neuroscience, Key Laboratory of Primate Neurobiology, CAS Center for Excellence in Brain Science and Intelligence Technology, Chinese Academy of Sciences, Shanghai, 200031 China; 2 Department of Child and Adolescent Psychiatry, Shanghai Mental Health Center, Shanghai Jiao Tong University School of Medicine, Shanghai, 200030 China; 3 Department of Physics, Shanghai Key Laboratory of Magnetic Resonance, East China Normal University, Shanghai, 200062 China; 4 Department of Psychological Medicine, Shanghai General Hospital, Shanghai Jiao Tong University School of Medicine, Shanghai, 200080 China

## Abstract

Hyperactivity of limbic (e.g., amygdalar) responses to negative stimuli has been implicated in the pathophysiology of generalized anxiety disorder (GAD). Evidence has also suggested that even a simple cognitive task involving emotionally salient stimuli can modulate limbic and prefrontal neural activation. However, whether neural modulation of emotional stimulus processing in a cognitive task is defective in adolescents with GAD has not yet been investigated. In this study, 20 adolescents with GAD and 14 comparable healthy controls underwent event-related functional magnetic resonance imaging (fMRI) coupled with an emotional valence evaluation task. During the evaluation of negative versus neutral stimuli, we found significant activation of the right inferior frontal gyrus (IFG) in healthy controls, while the bilateral amygdala was activated in GAD patients. Between-group analyses showed dramatically reduced task-activation of the right IFG in GAD patients, and the magnitude of IFG activity negatively correlated with symptom severity. Psychophysiological interaction analysis further revealed significantly decreased functional interaction between right IFG and anterior cingulate cortex and ventromedial prefrontal cortex in GAD patients compared with healthy controls. Taken together, our findings show failure to suppress negative affect by recruiting a cognitive distraction in adolescents with GAD, providing new insights into the pathophysiology of GAD.

## Introduction

Generalized anxiety disorder (GAD) commonly first emerging during childhood and adolescence, is intrinsically characterized by uncontrollable worry, hyper-vigilance, and excessive fear in multiple domains^1–4^. Adolescent GAD is a particularly high risk for adult anxiety^5^, suicidal ideation, and suicide attempt^6^. Given its prevalence and perniciousness in the adolescent population, there is a pressing need to advance understanding of GAD pathophysiology.

Functional magnetic resonance imaging (fMRI) coupled with overt symptom provocation paradigm has been widely used to examine emotion-related circuitry and elucidate the pathophysiology of GAD. Abnormal functional activation in the limbic system, which is responsible for the processing of negative affect input, has been reported in adolescents with GAD, often revealing pathologically exaggerated responses in the amygdala^7, 8^ (for reviews, see refs 2 and 9). Hyperactivity triggered by negative emotional stimuli has also been observed in the insula^3, 10^, another vital component of the core limbic system^11^, in individuals with anxiety disorders. However, most previous studies used emotional face stimuli, and subjects were instructed to passively view pictures in the absence of a cognitive task^2, 12^.

To better characterize the uncontrollable and diffuse anxiety experienced by GAD patients, Strawn and colleagues utilized an attention-demanding task with different non-face probes as emotional and neutral distractors to reveal dysfunction in additional circuits^13^. They identified increased activation in both the medial prefrontal cortex and ventrolateral prefrontal cortex in response to visual stimuli with emotional content^13^, further expanding the candidate regions of heuristic emotion circuits that may constitute the pathological basis of GAD. Moreover, neuroimaging studies have demonstrated the interactions between emotion and cognition by mapping the modulation of emotion-related regions by cognitive task. For example, Hariri *et al*.^14^ showed that labeling (compared to matching) angry or frightened expressions induces a diminished regional cerebral blood flow (rCBF) response in the bilateral amygdala with a simultaneous increase in rCBF in the right prefrontal cortex. There is also emerging evidence that rating the subjective experience of an aversive visual stimulus can decrease limbic activation and increase activity in the medial frontal regions^15^. These studies consistently suggest that even a simple cognitive task performed on emotionally salient stimuli can modulate neural activation in the limbic system and prefrontal cortex. The manner in which these two systems interact has been become increasingly central to models of psychopathology^16, 17^, which include defective modulation of the prefrontal system and enhanced engagement of the limbic system^18–20^. However, whether neural modulation of cognitive performance by emotional stimuli is defective in adolescents with GAD has not yet been investigated.

To address this question, we asked both adolescents with GAD and healthy controls (HCs) to perform an emotional valence evaluation task and simultaneously collected fMRI data. We adopted various non-face stimulus images from the International Affective Picture System (IAPS, University of Florida, Gainesville, Florida), to examine the processing of multiple situations and stimuli. Emotional valence evaluation is a cognitive task that allows subjects to regulate their experience of emotionally salient stimuli, specifically aversive or fear stimuli. Our study aimed to determine whether different brain activation patterns were recruited in adolescents with GAD and HCs while performing the valence evaluation task. Moreover, the contrast of negative versus neutral stimuli is commonly used to assess brain activation^12, 13, 15, 21^. However, this assessment cannot reveal the dynamic neural response that follows a stimulus. We therefore conducted a time course analysis for each stimulus type (i.e., negative and neutral stimuli) to examine the neural dynamics in the regions of interest (ROIs) showing significant difference in brain activation between GAD patients and HCs. To further explore the interaction between cognition and emotion, we performed psychophysiological interaction (PPI) analysis^22, 23^ for ROIs showing significant between-group difference in the contrast of negative versus neutral stimulus evaluation. A psychophysiological interaction indicates that the contribution of one area to another changes significantly with the experimental context. We hypothesized that the cognitive evaluation process can suppress negative affect, associated with higher activity of prefrontal cortex and less activity in the amygdala, in healthy subjects, but not in adolescents with GAD. Moreover, we predicted that the functional interaction between the prefrontal cortex and limbic system may be disrupted during negative versus neutral stimulus evaluation in adolescents with GAD.

## Materials and Methods

### Participants

A total of 24 adolescents with GAD were enrolled via child and adolescent outpatient clinics at Shanghai Mental Health Center, and 16 healthy comparison subjects were recruited from local middle schools (demographic and clinical data are displayed in Table 1). All participants are right handed, aged from 13 to 18 years old, and matched for education level. Participants with a primary diagnosis of GAD confirmed separately by two experienced child and adolescent psychiatrists in light of DSM-IV criteria were initially recruited. The Mini International Neuropsychiatric Interview for Children and Adolescents (MINI-KID)^24^ was then used to exclude subjects with a history of a manic episode, obsessive-compulsive disorder, major depressive disorder, post-traumatic stress disorder, substance dependence, transient tic disorder, attention deficit and hyperactivity disorder, anorexia nervosa, or pervasive developmental disorder. Participants who had contraindications to MRI were also excluded. No GAD patients were engaged in any type of formal psychotherapy or under psychotropic medication during or before the study. The Screen for Child Anxiety Related Emotional Disorders (SCARED)^25^ was administered to evaluate anxiety symptom severity. Healthy subjects were also screened with the MINI-KID, and individuals with a history of psychiatric or neurological disorders were excluded. The protocol of this study was approved by the Ethics Committee of Shanghai Mental Health Center (No. 2013–02) and all experiments were performed in accordance with the relevant guidelines and regulations. All participants and their parents provided written informed consent. To ensure a sufficiently large number of corrected trials, participants (three GAD patients and two HCs) with poor task performance (correct rate <70%) were excluded from subsequent image analysis. One GAD patient was excluded due to incomplete data. Excessive head motion was controlled by restricting translation less than 3.0 mm or rotation less than 3.0° in any direction. No participants were discarded based on this criterion. Thus, 20 GAD adolescents and 14 HCs were included in the final analysis.

**Table 1:** Demographic and diagnostic data.

	GAD Patients (n = 20)	HCs (n = 14)	p value
Age (mean ± SD), y	15.7 ± 1.7	15.5 ± 1.7	0.67
Gender (Male/Female)	5/15	6/8	0.16^*^
Education (mean ± SD), y	9.4 ± 1.7	9.6 ± 1.6	0.62
Handedness	R	R	—
SCARED Scores (mean ± SD)	41.3 ± 9.0	13.6 ± 6.2	<0.001
Comorbid Diagnosis, n (%)			
Specific phobia	4 (20.0%)	—	—
Social phobia	3 (15%)	—	—
Agoraphobia	3 (15%)	—	—
Panic disorder	2 (10%)	—	—
Separation anxiety	2 (10%)	—	—
Oppositional defiant	1 (5%)	—	—

Note: GAD = generalized anxiety disorder, HCs = healthy controls, SD = standard deviation, y = years, SCARED = Screen for Child Anxiety Related Emotional Disorders, R = right. The p values were obtained using two-tailed two sample test or ^*^Pearson Chi-Square test.

### Experimental Design

**Figure 1:**
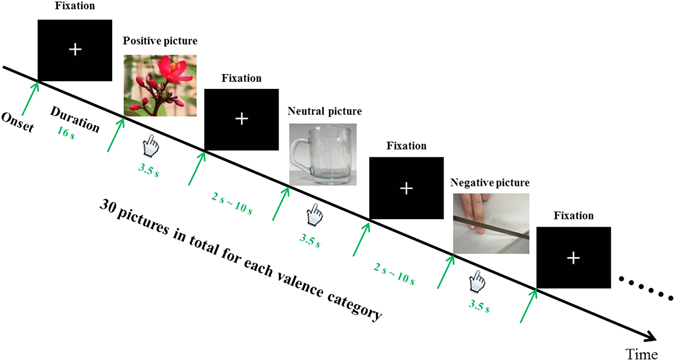
The experimental paradigm for the emotional valence-evaluation task. The task includes three valence categories of pictures (i.e., positive, neutral, and negative) and 30 pictures for each valence category. All pictures are presented for 3.5 s in a pseudorandom manner, followed by a fixation for 2 s, 4 s, 6 s, 8 s, or 10 s as an inter-stimuli-interval. Participants were asked to indicate the valence of the picture with a button-press action as quickly as possible. The subjects response to the different stimuli using the different fingers in two hands (i.e., positive, neutral, and negative stimuli corresponding to right index finger, right middle finger, and left index finger respectively). The emotional and neutral images presented are not from the International Affective Picture System, but rather, are representative images.

Participants were instructed to perform a valence evaluation task while viewing a variety of affective pictures (including positive, neutral, and negative emotional valences) during an fMRI scan. All pictures were sampled from the IAPS (University of Florida, Gainesville, Florida). The mean (SD) normative valence ratings were 7.94 (1.41), 4.99 (1.07), and 2.09 (1.36) for positive, neutral, and negative pictures, respectively. The mean (SD) normative arousal ratings were 4.73 (2.43), 3.03 (1.91), and 5.92 (2.22) for positive, neutral, and negative pictures, respectively. Each participant performed two runs in which a standard event-related paradigm was adopted, as illustrated in Fig. 1. Each run consisted of 45 trials, resulting in a total of 90 trials. For each trial, an affective image was presented for 3.5 s, followed by an eye fixation for 2 s, 4 s, 6 s, 8 s, or 10 s as a stimulus interval. Images from different valence categories were presented in a pseudorandom order (30 trials in total for each valence category). Participants were asked to evaluate the valence of the presented image with a button-press response as soon as possible. The subjects responded to different categories of stimuli using the different fingers of both hands (i.e., positive, neutral, and negative stimuli corresponded to the right index finger, right middle finger, and left index finger respectively). All the subjects practiced responding to the stimuli outside the MRI scanner before formal experiment. The presentation of visual stimuli and the recording of behavioral data (e.g., performance accuracy and reaction time) were done by E-prime software (Psychology Software Tools, Inc. Pittsburgh, PA, USA).

### MRI Data Acquisition

Imaging data were collected on a Siemens Trio 3.0 Tesla MRI scanner (Siemens, Erlangen, Germany) at the Shanghai Key Laboratory of Magnetic Resonance, East China Normal University. Task-based fMRI data covering the whole brain were acquired using a T2*-weighted EPI (echo-planar imaging) sequence: 38 axial slices, thickness = 4 mm, matrix = 64 × 64, repetition time = 2,000 ms, echo time = 21 ms, and field of view = 224 mm × 224 mm. Three-dimensional T1-weighted images were obtained in a sagittal orientation employing a MPRAGE (magnetization prepared rapid gradient echo) sequence: 192 slices per slab, thickness = 1 mm, repetition time = 2,530 ms, echo time = 2.34 ms, matrix = 224 × 256, and field of view = 224 mm × 256 mm.

### Preprocessing of fMRI Data

All fMRI data were analyzed using the Statistical Parametric Mapping package (SPM8, http://www.fil.ion.ucl.ac.uk/spm). Preprocessing of fMRI data was performed as follows: the images were first corrected for delay in slice acquisition and rigid-body head movement. The corrected images were subsequently spatially normalized to the MNI (Montreal Neurological Institute) space using a unified segmentation algorithm^26^ and then resampled to 3 mm isotropic voxels. Finally, spatial smoothing was conducted using an isotropic Gaussian filter at full width at a half maximum (FWHM) of 8 mm.

### Univariate Brain Activation Analysis

Individual activation maps were created using standard procedures in SPM8 software. We modeled each category of events (positive, neutral, and negative trials) with delta functions at the onset of events, convolved with a canonical hemodynamic response function. Each category of events was defined as one condition in the general linear model (GLM), with error trials and six parameters of head movement specified as regressors of no interest. The contrast maps of negative versus neutral were obtained based on beta values estimated from the GLM model, and subjected to group-wise activation analysis. The clustered regions that were robustly and consistently activated were determined by p < 0.05 with AlphaSim corrected (i.e., p-voxel < 0.0005 with minimum cluster size = 22 voxels). Between-group analysis was further performed using two-tailed two sample t test, with age, gender, and education as covariates. We considered p < 0.05, AlphaSim corrected (i.e., p-voxel < 0.001 with minimum cluster size = 12 voxels) as statistically significant. Notably, the multiple comparison correction for the two sample t test was conducted within a small volume, obtained by combining the within-group activation maps. To further test the relationship between neural response and clinical anxiety symptoms, we examined correlations between magnitude of brain activity in the regions showing significant between-group differences and SCARED scores.

### Time Course Analysis

To explore dynamic neural response during emotional valence evaluation, we performed the time course analysis as follows. The preprocessed BOLD (blood oxygenation level dependent) signal was extracted for each stimulus type (i.e., negative and neutral stimuli). An average of the BOLD signal for all of the voxels within each ROI was plotted to represent the experimentally derived hemodynamic response function during each stimulus condition. For the ROIs, we mainly focused on the regions showing significant activation differences in response to negative versus neutral stimuli evaluation between GAD patients and HCs. The average BOLD signal values were then converted to percent signal change relative to the average of stimulus onset points. Here, we plotted 6 time points (i.e., 6TR = 12 s) following stimulus onset for each stimulus condition. The differences of percent BOLD signal change between adolescents with GAD and HCs were evaluated using a two-tailed two sample t test at each time point, with age, gender, and education as covariates. We considered p < 0.05 to be statistically significant.

### Psychophysiological Interaction Analysis

To further explore brain interactions in response to negative versus neutral stimuli, we performed a PPI analysis^22, 23^ to assess context-dependent functional connectivity. We utilized established procedures in SPM8 software for PPI analysis. Briefly, we first identified a spherical ROI with its origin at the MNI coordinate of peak t-score (derived from between-group difference of task activation) and a radius equal to 6 mm as the seed or source region. Secondly, the first eigenvariate time series of the BOLD signal from the ROI (derived from the contrast of negative versus neutral stimulus evaluation) was extracted for each subject. A deconvolution step was then applied in the first eigenvariate time series with a HRF. Thirdly, the PPI interaction term was calculated as the product of the time series of the seed ROI (physiological factor) and the vector coding for the task factor (psychological factor). Finally, we performed a PPI GLM analysis that includes the interaction term, ROI signal, and the experimental vector in the design. The resulting SPM exhibits areas showing differential connectivity to the ROI due to the effect of negative versus neutral conditions. The within-group connectivity pattern of PPI was obtained by one sample t test with a threshold of p < 0.05 with AlphaSim correction (i.e., p-voxel < 0.0005 with minimum cluster size = 22 voxels). Between-group analysis was further performed using a two-tailed two sample t test, with age, gender, and education as covariates. We considered p-voxel < 0.001 (uncorrected) as statistically significant.

## Results

### Behavioral Data

Two behavioral parameters, performance accuracy and reaction time, were recorded during the task and compared between GAD patients and HCs. We found no significant difference in correct rates for evaluation of negative (p > 0.1), neutral (p > 0.1), or positive (p > 0.1) pictures. With regard to reaction time, we observed no significant difference between the two groups for the evaluation of positive (p > 0.1) or negative (p > 0.1) pictures. However, the reaction time for evaluation of neutral pictures of the GAD group was enhanced compared to the healthy group (p = 0.04).

### Brain Activation Pattern Response to Evaluation of Negative versus Neutral Stimuli

**Figure 2:**
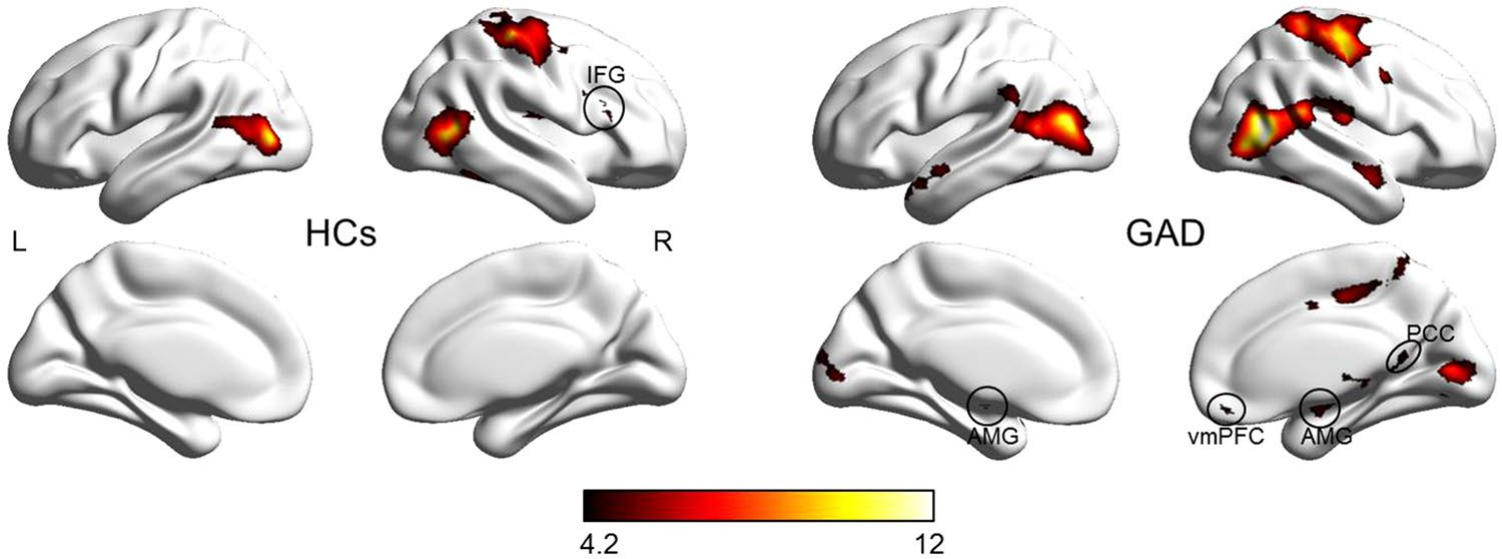
Within-group brain activation for the contrast of negative versus neutral stimuli evaluation in adolescents with GAD and HCs. The threshold was set at p < 0.05, AlphaSim corrected (i.e., p-voxel < 0.0005 with minimum cluster size = 22 voxels). Color bar denotes t value. IFG = inferior frontal gyrus, AMG = amygdala, vmPFC = ventromedial prefrontal cortex, PCC = posterior cingulate cortex, L = left, R = right, GAD = generalized anxiety disorder, and HCs = healthy controls.

**Figure 3:**
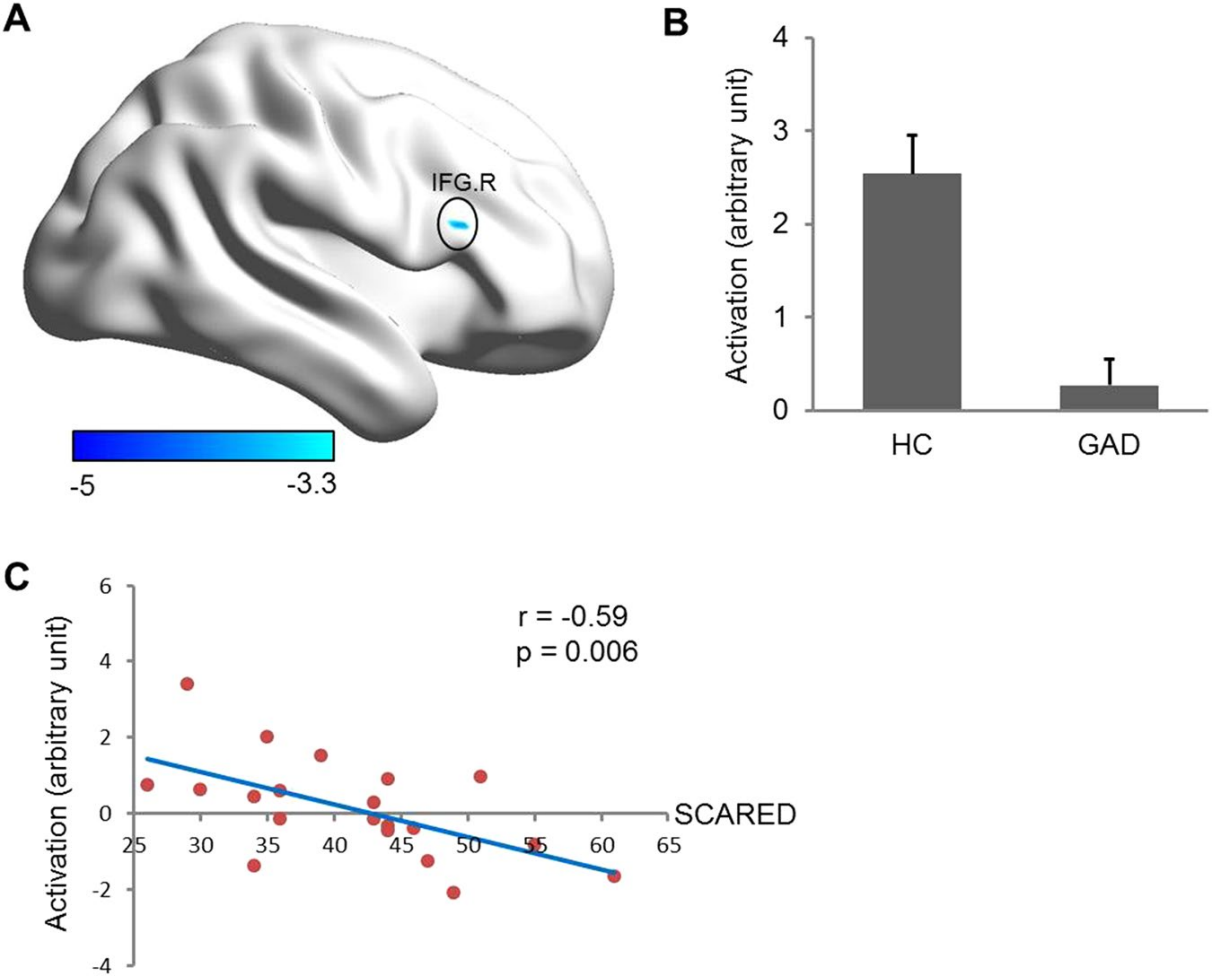
(**A**) Significantly decreased brain activation for the contrast of negative versus neutral in adolescents with GAD compared with HCs. We considered p < 0.05, AlphaSim corrected (i.e., p-voxel < 0.001 with minimum cluster size = 12 voxels) to be statistically significant. Color bar denotes t value. (**B**) Histogram shows the activation magnitude of right IFG in GAD patients and HCs. Error bar denotes the SEM. (**C**) Significant correlation was observed between activation magnitude of right IFG and SCARED scores in GAD patients. SCARED = Screen for Child Anxiety Related Emotional Disorders, IFG = inferior frontal gyrus, R = right, GAD = generalized anxiety disorder, and HCs = healthy controls.

During the evaluation of negative versus neutral stimuli, we found the right inferior frontal gyrus (IFG), insula, middle temporal gyrus (MTG), middle occipital gyrus (MOG), and sensorimotor area to be significantly activated in HCs; in contrast, adolescents with GAD exhibited activation in the bilateral amygdala/hippocampus, ventromedial prefrontal cortex (vmPFC), posterior cingulate cortex (PCC), insula, thalamus, MTG, MOG, and sensorimotor area (Fig. 2 and Table 2). In between-group analysis, we observed significantly decreased activation of the right IFG in response to the evaluation of negative versus neutral stimuli in adolescents with GAD compared to HCs. Moreover, the magnitude of right IFG activity negatively correlated with severity of anxiety symptoms in adolescents with GAD (r = −0.59, p = 0.006) (Fig. 3).

**Table 2:** Brain activation in response to negative versus neutral stimulus processing.

Brain Regions	MNI Coordinates			Peak t-score	Number of voxels	Volume (mm^3^)
	x	y	z			
**Within healthy control group**						
Inferior Frontal Gyrus (R)	57	27	18	6.1	60	1620
Insula (R)	36	−12	15	5.5	50	1350
Middle temporal gyrus/middle occipital gyrus (R)	48	−60	9	10.0	224	6048
Fusiform gyrus (L)	−39	−45	−15	6.7	25	675
Middle occipital gyrus/middle temporal gyrus (L)	−48	−78	3	10.9	115	3105
Sensorimotor area (R)	42	−30	60	10.2	778	21006
**Within GAD patient group**						
Amygdala/hippocampus (R)	24	−4.8	−15	5.9	52	1404
Amygdala/hippocampus (L)	−21	−9	−18	5.3	46	1242
Insula (R)	34	−18	15	9.1	121	3267
Medial prefrontal cortex (R)	9	45	−18	5.3	25	675
Dorsomedial frontal cortex (L)	−3	54	24	4.6	48	1296
Posterior cingulate gyrus (R)	9	−51	12	4.5	41	1107
Middle cingulate gyrus (R)	3	0	42	5.9	41	1107
Thalamus (R)	12	−18	6	6.3	86	2322
Middle temporal gyrus/middle occipital gyrus (R)	54	−63	3	14.2	472	12744
Middle occipital gyrus/middle temporal gyrus (L)	−48	−75	12	12.1	208	5616
Fusiform gyrus (L)	−42	−45	−18	6.6	62	1674
Fusiform gyrus (R)	42	−39	−21	6.3	64	1728
Sensorimotor area (R)	39	−18	57	10.3	895	24165
**GAD versus control groups**						
Inferior Frontal Gyrus (R)	48	18	15	−5.1	21	567

Note: GAD = generalized anxiety disorder, L = left, R = right. For the group-wise analysis, the threshold was set at p < 0.05, AlphaSim corrected (i.e., p-voxel < 0.0005 with minimum cluster size = 22 voxels). For the Between-group analysis, we considered p < 0.05, AlphaSim corrected (i.e., p-voxel < 0.001 with minimum cluster size = 12 voxels) to be statistically significant.

### Dynamic Neural Response for the Evaluation of Negative and Neutral Stimuli

**Figure 4:**
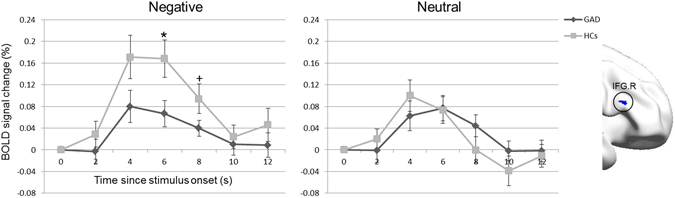
Dynamic neural response in the right IFG during negative and neutral stimuli evaluation. IFG = inferior frontal gyrus, GAD = generalized anxiety disorder, HCs = healthy controls, and R = right. Error bar denotes the SEM. ^*^p < 0.05, ^+^p = 0.05.

We found the percent BOLD signal change in the right IFG response to the evaluation of negative stimuli to be significantly lower in the GAD patients than HCs, with this difference reaching a statistically significant level (p = 0.019) at 6 s post-stimulus. However, the dynamics of signal change were synchronized. In contrast, the percent BOLD signal change response to evaluating neutral stimuli peaked later in GAD patients than in HCs, at a similar peak value (Fig. 4).

### Psychophysiological Interaction Analysis

**Figure 5:**
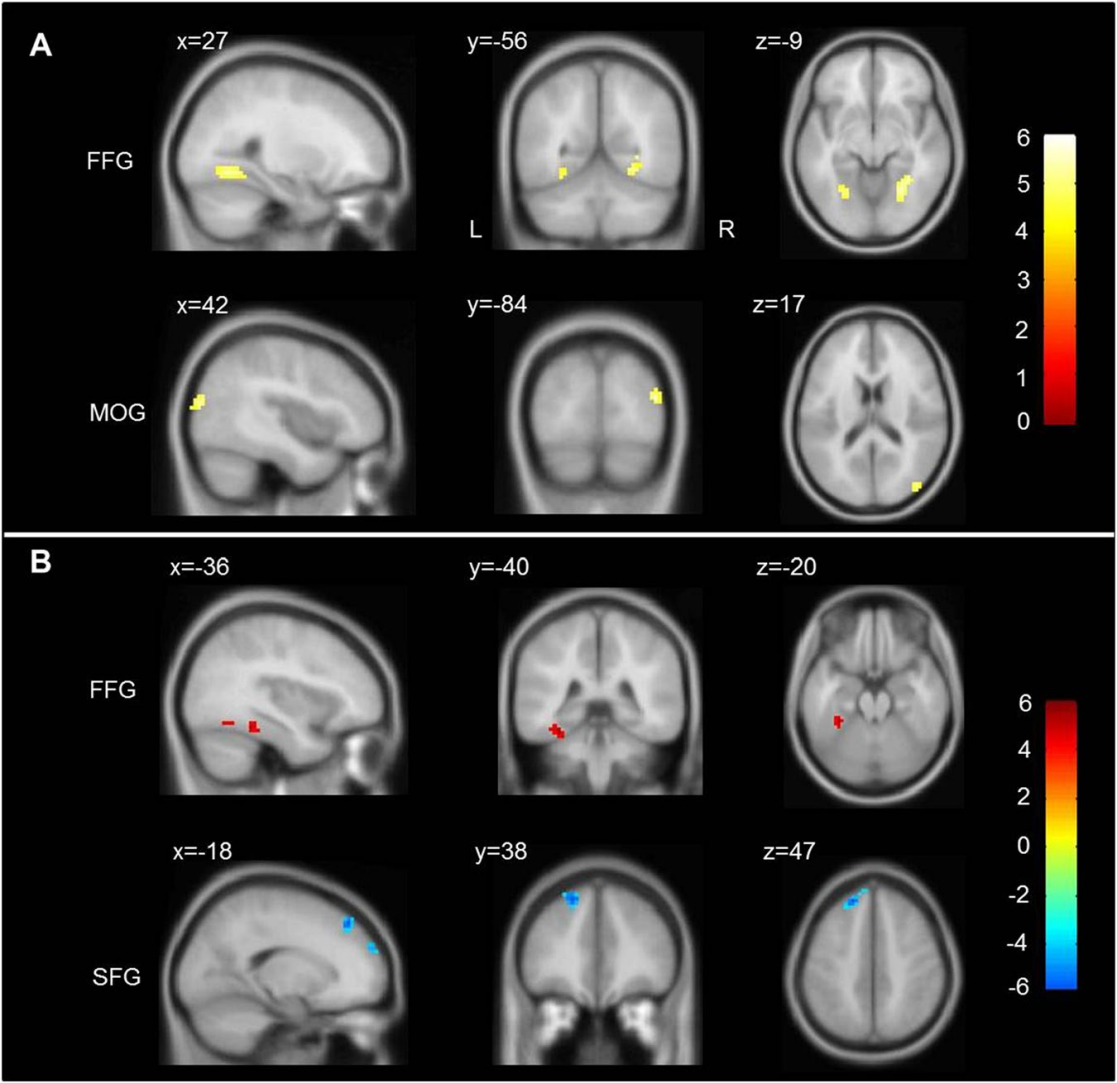
Regions showing significant psychophysiological interaction (PPI) with right IFG during negative versus neutral stimuli evaluation in HCs (**A**) and GAD patients (**B**). The threshold was set at p < 0.05, AlphaSim corrected (i.e., p-voxel < 0.0005 with minimum cluster size = 22 voxels). Color bar denotes t value. x, y, z denote MNI (Montreal Neurological Institute) coordinates. IFG = inferior frontal gyrus, FFG = fusiform gyrus, MOG = middle occipital gyrus, SFG = superior frontal gyrus, GAD = generalized anxiety disorder, HCs = healthy controls, L = left, and R = right.

**Figure 6:**
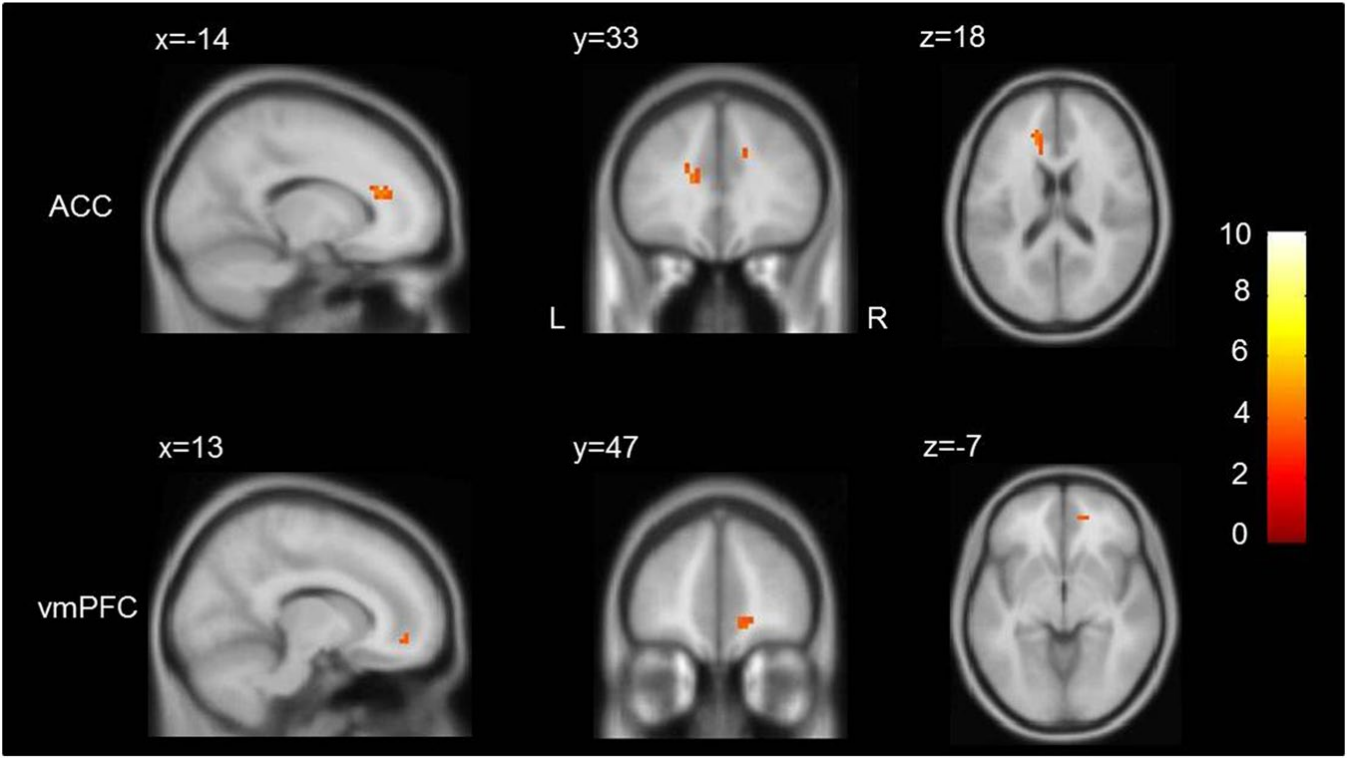
Regions showing reduced psychophysiological interaction (PPI) with right IFG during negative versus neutral stimuli evaluation in adolescents with GAD compared with HCs. The threshold was set at p < 0.001 (uncorrected). Color bar denotes t value. x, y, z denote MNI (Montreal Neurological Institute) coordinates. IFG = inferior frontal gyrus, ACC = anterior cingulate cortex, vmPFC = ventromedial prefrontal cortex, GAD = generalized anxiety disorder, HCs = healthy controls, L = left, and R = right.

To explore brain interactions, we further conducted a PPI analysis for the right IFG in the contrast of negative versus neutral stimuli evaluation. For the within-group analysis, we found positively functional coupling between the right IFG and bilateral fusiform gyri and right MOG in HCs. In contrast, we observed that the right IFG positively interacted with the left fusiform gyrus, and negatively interacted with the superior frontal gyrus in GAD patients (Fig. 5 and Table 3). Compared with HCs, we found that functional interaction between right IFG and anterior cingulate cortex (ACC) and vmPFC was significantly decreased in adolescents with GAD (Fig. 6 and Table 3). However, we did not observe any correlation between functional interaction and clinical scores.

**Table 3:** Regions showing significant psychophysiological interaction (PPI) with right inferior frontal gyrus in the contrast of negative versus neutral stimulus processing.

Brain Regions	MNI Coordinates			Peak t-score	Number of voxels	Volume (mm^3^)
	x	y	z			
**Within healthy control group**						
Fusiform gyrus (L)	−24	−63	−12	5.4	25	675
Fusiform gyrus (R)	33	−51	−9	5.6	45	1215
Middle occipital gyrus (R)	42	−84	15	5.7	22	594
**Within GAD patient group**						
Fusiform gyrus (L)	−33	−39	−21	5.1	41	1107
Superior frontal gyrus (L)	−18	39	48	−5.7	56	1512
**GAD versus control groups**						
Anterior cingulate gyrus (L)	−15	30	18	−4.5	18	486
Anterior cingulate gyrus (R)	18	27	30	−3.9	9	243
Ventromedial prefrontal gyrus (R)	15	48	−9	−4.0	7	189

Note: GAD = generalized anxiety disorder, L = left, R = right. For the group-wise analysis, the threshold was set at p < 0.05, AlphaSim corrected (i.e., p-voxel < 0.0005 with minimum cluster size = 22 voxels). For the Between-group analysis, we considered p-voxel < 0.001(uncorrected) to be statistically significant.

## Discussion

This study investigated neural modulation of a simple cognitive task (i.e., evaluation of emotional valence) operating upon salient emotional stimuli in both adolescents with GAD and HCs. For the within-group activation analysis, we found the right IFG to be significantly activated during the evaluation of negative versus neutral stimuli in HCs. In contrast, the bilateral amygdala and default mode regions (i.e., vmPFC and PCC) were activated in adolescents with GAD. Two brain systems are commonly used to model emotion processing^17, 19^. The ventral system primarily encompasses the amygdala, and is involved in the rapid appraisal of emotion-related stimuli and generation of affective response, while the dorsal system is mainly comprised of prefrontal regions and is responsible for emotion regulation^27–30^. Recent research has become increasingly focused on the interaction between these two systems. Previous studies in healthy subjects have demonstrated that even a simple cognitive task, such as rating^15^ or labeling^14^, performed on emotionally salient stimuli can reduce activation of the amygdala and simultaneously increase activity in the prefrontal cortex. It is possible that cognitive demands have reduced resources available for the processing of emotional content, thus suppressing the emotional response. Our findings consistently indicate that the amygdala-based associative level of emotion processing is subject to cognitive modulation by the prefrontal cortex in HCs, but not in GAD patients. This result has, on one hand, confirmed that the paradigm (i.e., emotional valence evaluation task) used in this study is suitable for investigating interactions between cognition and emotion, and on the other hand suggests that healthy subjects are able to successfully suppress negative affect by cognitive distraction, while adolescents with GAD are not.

It is worth noting that different cognitive tasks may recruit different parts of the prefrontal cortex; for example, rating increases activity of the medial prefrontal cortex, and labeling increases activity of the right lateral prefrontal cortex in healthy subjects^14, 15^. In contrast, our results indicate that the right IFG is significantly activated in response to the evaluation task in HCs, but not in GAD patients. Moreover, the magnitude of right IFG activity negatively correlates with anxiety symptom severity. The right IFG is considered to play a crucial role in attention control^31, 32^. Specifically, during the dual-task interference experiment, the right IFG is recruited to suppress the second task until processing resources are liberated^33^. A previous study also reported that cognitive control of anxiety state from threat-related distractors was associated with IFG activation and simultaneously decreased amygdala activation^34^. Moreover, IFG activation has been related to better working memory performance during emotional distraction^35^. We therefore speculate that the right IFG is involved in shifting attention from emotional experience to cognitive task execution. Thus, decreased right IFG activity may result in the defective suppression of emotional response in GAD patients.

Unexpectedly, we observed that default mode regions (i.e., vmPFC and PCC) were activated throughout the evaluation of negative versus neutral stimuli only in GAD patients. The role of the default mode network (DMN) in self-reference is well established^36–38^. Although the DMN is more active at rest than during tasks, it can be modulated by emotional state^39, 40^ or cognitive load of an active task^41^. It has been reported that social phobia patients show abnormal activity in the DMN network, which may be attributable to self-focused attention^42^. Self-focused attention is an increased awareness of self-referential information, and is present in many affective disorders, such as social phobia and social anxiety^43^. Accordingly, DMN activation in GAD patients may be related to self-focused attention, leading to increased access to negative feelings. Such findings reiterate the pivotal role of aggravated attention bias toward internal threat in characterizing psychopathological domains of anxiety disorders, separate from the component of excessive fear^44, 45^.

Through dynamic time course analysis, we found a synchronized dynamic neural response in the right IFG between GAD patients and HCs during negative stimuli evaluation, although the magnitude of percent BOLD signal change was lower in the GAD sample. In contrast, the percent BOLD signal change in the right IFG response to evaluating neutral stimuli peaked later in the adolescents with GAD compared to HCs. Behaviorally, we observed significantly increased reaction time in GAD patients for neutral picture evaluation, but not for negative stimulus evaluation. Due to low arousal level, it is more difficult to evaluate neutral than negative stimuli. Furthermore, pathological worry, the hallmark of GAD, has been linked to deficits in executive processes^46^. It is possible that delayed neural response in the right IFG during neutral stimuli evaluation underlies the observed prolonged reaction time.

In addition to different activation areas in HCs and GAD patients, we also observed commonly activated regions in the two groups during negative versus neutral stimulus processing, such as insula, MTG, MOG, and sensorimotor area. Although the insula activated in the both two groups, there is a larger extent of activation in GAD patients. The insula putatively codes interoceptive representation and plays a pivotal role in processing the subjective experience of aversive stimuli^47, 48^. The subjective experience of emotional stimuli is indeed required to perform our evaluation task. The alteration of interoceptive states in anxiety disorders is hypothesized to be a consequence of noisy amplification of the self-referential interoceptive prediction signal^47, 49^. MTG and MOG are high-level visual areas and play important roles in perceiving and recognizing objects^50, 51^. Moreover, evidence from fMRI and neurophysiological studies suggest that emotional (especially fearful) stimuli can enhance activity of the visual system responsible for object recognition and presumably through direct feedback connections from the amygdala^52^. Accordingly, we found dramatic activation of MTG and MOG during the processing of negative relative to neutral stimuli in both HCs and GAD patients. This result not only confirms previous findings, but also suggests that the emotional modulation of perception is intact in adolescents with GAD. The activation of the sensorimotor area likely occurs because the motor responses to negative and neutral stimuli require the use of fingers in different hands.

Using PPI analysis, we found that the right IFG positively interacted with high-level visual regions (e.g., fusiform) in both HCs and GAD patients during negative versus neutral stimuli evaluation. A previous study combining magnetoencephalography and fMRI has indicated that the inferior frontal junction may direct the flow of visual processing during attention to objects and their features^53^. Moreover, many behavioral observations indicate that people more readily pay attention to emotional than neutral stimuli, and that this occurs in a reflexive and involuntary manner^54^. It is likely that functional interaction between the right IFG and high-level visual system contributes to the emotional salience of objects. In addition, we observed a negative interaction between right IFG and superior frontal gyrus within GAD patients during negative relative to neutral stimuli evaluation. As mentioned above, GAD patients show increased reaction time and delayed neural response in the right IFG for neutral stimuli evaluation, suggesting increased cognitive effort. We therefore propose that the negative functional interaction may play a compensatory role in evaluation of neutral stimuli in GAD patients.

We further observed significantly reduced functional interaction between right IFG and ACC and vmPFC in adolescents with GAD compared to HCs. Many neuroimaging studies have indicated that ACC and vmPFC are key regions for emotional regulation^11, 17, 55^. In particular, one previous study observed a significant focus of activation in ACC during the condition requiring attention to subjective emotional responses, but not during the condition requiring attention to stimulus context^56^. A recent study also indicates increased top-down attentional control causes increased connectivity between dorsolateral PFC and dorsal ACC^19^. This suggests the ACC plays a crucial role in attentional processing^57^. It is possible that cognitive modulation of emotion responses is by ACC not directly on amygdala. Moreover, reduced structural integrity of uncinate fasciculus, a major white matter tract that directly connects the amygdala and ACC and ventral PFC, has been detected in GAD patients^58^. Therefore, we speculate that disrupted functional interaction between IFG and limbic system, such as ACC, may lead to failure in cognitive suppression of negative affect in adolescents with GAD.

This study must be interpreted in the context of its limitations. We cannot completely rule out potential variations and confounds caused by comorbidity^2, 44, 59^, although we did conduct rigorous screening during patient recruitment. In addition, previous studies in healthy subjects have performed rating and labeling tasks on emotionally salient stimuli, with passive view as a control experiment^14, 15^. In the current study, a passive task was not carried out, to reduce scanning time for the teenage subjects. Additionally, our study focused on the altered neural interactions between cognition and emotion in adolescents with GAD. Further caution is warranted in the interpretation of our results in relation to findings reported in adult GAD, as developmental factors can confound the present protocol. Finally, the subjects in this study are all adolescents with relatively poor motion restraint during scanning. In order to include more subjects, we used a relatively loose criterion (i.e., less than 3.0 mm or rotation less than 3.0° in any direction) for defining excessive head motion. Although we have considered the 6 head motion parameters as covariates in the GLM to control the potential impact of motion, we also performed a correlation analysis to further examine the influence of head motion on our main findings. As a result, we did not find any correlations between mean displacement of motion and activation of right IFG or SCARED score. This finding suggests that our main results are not affected by slight head motion. Despite these limitations, our current findings indicate defective interactions between cognition and emotion in adolescents with GAD, providing theoretical guidance for the improvement of cognitive behavioral treatments targeting such deficits in young patients.
